# Recent Developments in Systems Biology and Metabolic Engineering of Plant–Microbe Interactions

**DOI:** 10.3389/fpls.2016.01421

**Published:** 2016-09-26

**Authors:** Vishal Kumar, Mehak Baweja, Puneet K. Singh, Pratyoosh Shukla

**Affiliations:** Enzyme Technology and Protein Bioinformatics Laboratory, Department of Microbiology, Maharshi Dayanand UniversityRohtak, India

**Keywords:** plant–microbe interactions, signaling, systems biology, CRISPR-Cas, gene editing

## Abstract

Microorganisms play a crucial role in the sustainability of the various ecosystems. The characterization of various interactions between microorganisms and other biotic factors is a necessary footstep to understand the association and functions of microbial communities. Among the different microbial interactions in an ecosystem, plant–microbe interaction plays an important role to balance the ecosystem. The present review explores plant–microbe interactions using gene editing and system biology tools toward the comprehension in improvement of plant traits. Further, system biology tools like FBA (flux balance analysis), OptKnock, and constraint-based modeling helps in understanding such interactions as a whole. In addition, various gene editing tools have been summarized and a strategy has been hypothesized for the development of disease free plants. Furthermore, we have tried to summarize the predictions through data retrieved from various types of sources such as high throughput sequencing data (e.g., single nucleotide polymorphism detection, RNA-seq, proteomics) and metabolic models have been reconstructed from such sequences for species communities. It is well known fact that systems biology approaches and modeling of biological networks will enable us to learn the insight of such network and will also help further in understanding these interactions.

## Introduction

Microbial interactions have a decisive role in the sustainability of the various ecosystems. The characterization of such interactions among microorganisms and other biotic factors is a necessary footstep to understand the association and functions of microbial communities. Among the different microbial interactions in an ecosystem, plant–microbe interaction plays an important role to balance the ecosystem. Plants produce a number of organic and inorganic compounds which results in a nutritionally enriched environment which is favorable for heavy colonization of diversity of microbes. Microorganisms may colonize the exteriorly (epiphytes) or interiorly (endophytes). Microbial communities can affect the plant physiology either positively or negatively in direct or indirect ways by various interactions mutualism, commensalism, amensalism, and pathogenic consequences. Endophytic bacteria is an example of plant–microbe interaction wherein bacteria live in a non-competitive environment of host plant tissue without any major damage to the host cell (James and Olivares, 1998). Endophytes are omnipresent in nearly all plants on earth. Endophytic microflora such as bacteria and fungi, are defined as microorganisms which are present after surface sterilization of various plant parts such as root, shoot, seed, or nodules. It has been assumed that these endophytes originated from the rhizosphere, the seeds, and the aerial portion of plants (Seghers et al., 2004). The rhizospheric soil is a significant source of root endophytes (Gao et al., 2004; Castro-Sowinski et al., 2007; Imam et al., 2013a). These endophytic microbes are supposed to enter into the plant tissue by local fractures or cellulose degradation of the root system (Gough et al., 1997). Endophytes inside a plant tissue may either be restricted to the point of entry or extend throughout the plant. These bacteria generally colonize the intercellular spaces, and they have been isolated from all compartments including seeds. There are few studies on plant–microbe interactions on details about Avr protein, computational strategies for protein interactions, molecular diversity and interactions of virulence genes (Imam et al., 2013a,b,c, 2014, 2015a,b). Both types of bacteria either Gram-positive or Gram-negative have been isolated from different tissues of numerous types of plant species. A number of facultative endophytes have been reported from rice, maize, wheat, sorghum, cotton, potato, and *Arabidopsis*. Furthermore, several different bacterial species have been isolated from a single plant. Conventionally, to investigate the various plant–microbial interactions use of a number of laborious laboratory experiments such as growth assays and pot house experiments are required (Kato et al., 2005; Harcombe, 2010; Zeidan et al., 2010). However, these laborious experiments make them infeasible for large scale application. With the help of bioinformatics approaches these issues can be alleviated by predicting plant–microbe interactions for experimental validation (Freilich et al., 2011; Buffie et al., 2014; Lima-Mendez et al., 2015). These predictions are founded on different types of informational data, such as the measurement of species abundances from high throughput sequencing or reconstructed metabolic models for species communities. There are several reports in various related fields where use of gene editing, genome engineering, and advanced technologies are proving quite significantly addressed (Gupta and Shukla, 2015a,b, 2016). In addition, various other *in silico* methods could be relevant to analyze such interactions while understanding the large amount of published data (Pritchard and Birch, 2011; Xu et al., 2013; Dix et al., 2016). This review envisages the concept of systems biology and gene editing in plant–microbe interactions by deciphering these technologies in detail.

## Plant–Microbe Interaction and its Relevance

**FIGURE 1:**
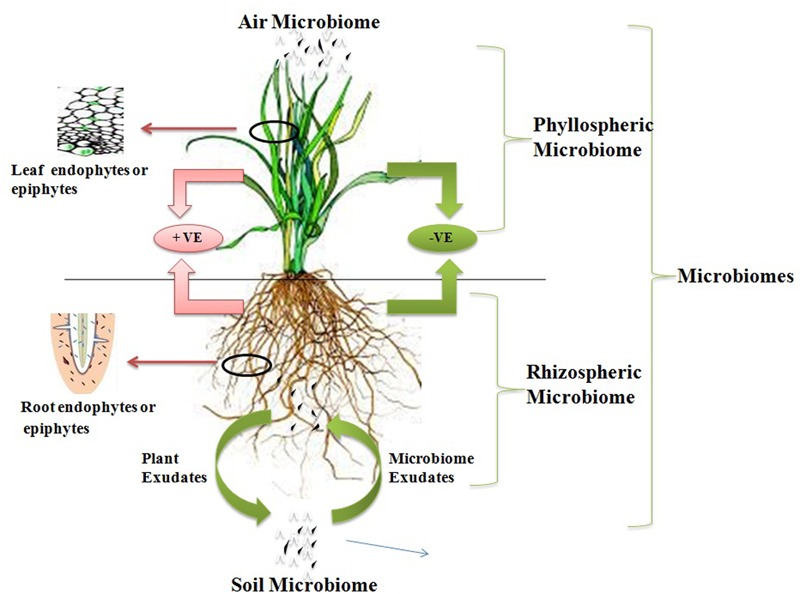
**Representation of plant–microbe interaction in different microbiome**.

Microflora is an aggregation of several types of microbes to form heterogeneous communities which are necessary components in several ecological niches and composed of distinct proportions of various microorganisms. Microorganisms of microflora do not live isolated or independently, but in its place these populations actively interact with other biological members of the ecosystem within their ecological niche. These microbial interactions may take place with any of biological form such as animal–microbe interaction, microbe–microbe interaction, plant–microbe interaction, etc. Plants provide an excellent ecosystem for microbial interactions. The plant provides the variable environment to the microorganisms from aerial plant part to the stable root system for the interactions. On the basis of location of plant–microbe interaction, the microbes can be divided in two groups, phyllospheric microorganisms which interact with the aerial leaf surface of plants and rhizospheric which interact with roots of plants. Phyllospheric microorganisms are adapted to low humidity and high irradiation, helps to protect plants from airborne pathogens. Rhizosphere of plants is a nutritionally rich zone due to deposition of nutrition rich compounds such as amino acid, organic acid, vitamins, sugars, etc. secreted by the roots. There is a pictorial presentation of various microbiome in **Figure 1** showing both phyllospheric and rhizospheric microorganisms. The nutritional enriched environment around roots creates a favorable environment for the growth of soil microorganisms, which includes rhizosphere and the rhizoplane soil microbial communities. A number of microorganisms interact with different plant tissues or cells with various level of dependence. These interactions may be beneficial, harmful, or neutral for one or both the organisms on the basis of this attribute plant–microbe interactions are known as amensalism (neutral–negative), antagonism (negative–positive), commensalism (neutral–positive), competition (negative–negative), mutualism (positive–positive), and neutralism (neutral–neutral). The commensalism or mutualism are more frequent interactions found in plants, in which either one or both species gain benefit from the relationship respectively (Campbell, 1995). Mycorrhiza and genus *Rhizobium* symbionts are best example of mutualism interaction. There are a number of superb reviews reporting present research on plant–microbe interaction at the molecular level, plant responses to quorum-sensing signals from microbial communities, applications of plant–microbe interaction, microflora responses toward transgenic plants and other rhizospheric interactions (Bauer and Mathesius, 2004; Singh et al., 2004; Sørensen and Sessitsch, 2007; Fillion, 2008; Ryan et al., 2008). The examination and understanding of these plant–microbe interactions helps to figure out the insights of mechanism which may direct us to understand such concerns. These sustainable resources will be ecofriendly and helpful to clean up the pollution and gaseous effect on a global scale.

## Systems Biology Approaches in Plant–Microbe Interactions

### Communication Systems

**FIGURE 2:**
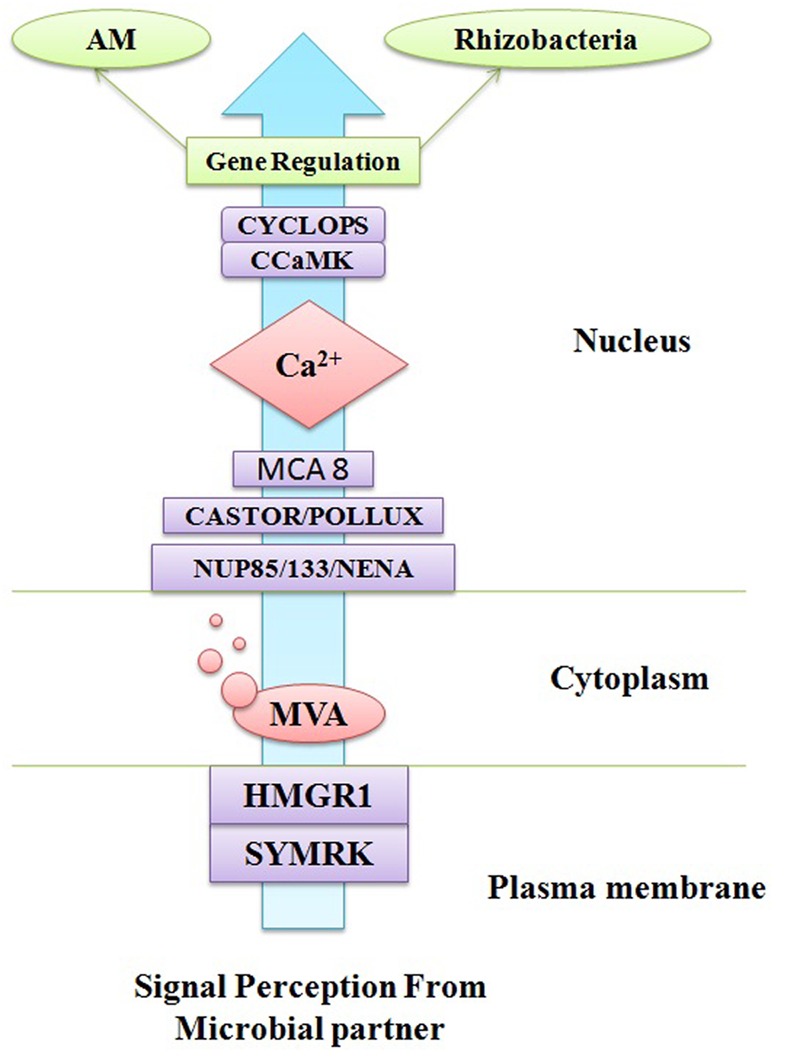
**A common signaling pathway triggers in plant cell during microbial interaction**.

**FIGURE 3:**
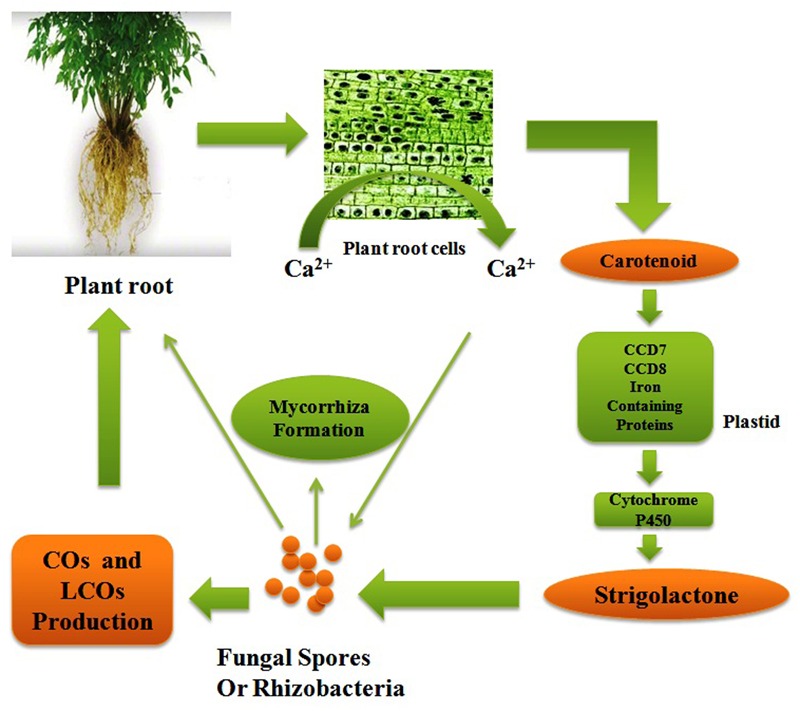
**A Signal communication system between plant root and arbuscular fungi**.

The life cycles of all the organisms from quorum sensing bacteria (Cornforth et al., 2014) to singing whales (Parks et al., 2014) are found on signaling pathways to convey information. Signaling system has played an important role in organismal evolution and the complexity of life (West et al., 2015). If both the donor as well as a receiver has a shared interest to propagate the reliable information then an effective signaling system can fetch a number of health benefits. The signaling pathway may be important from an evolutionary point of view because organisms can manipulate signals for their profit (Mokkonen and Lindstedt, 2015). Now these days, there has been an escalating awareness in communication network between the plants and root microflora which have a symbiotic relationship (Miller and Oldroyd, 2012: Bakker et al., 2013; Andreo-Jimenez et al., 2015). The roots of plant are bordered by a massive amount of soil microorganisms consisting of tens of thousands species diversity (Bardgett and van der Putten, 2014). There should be an effective crosstalk between plant and surrounding microflora to establish a successful relationship. There should a better understanding of these molecular signaling pathways to access control over the microbial population. The researchers have made efforts from last decade to understand the molecular mechanism of communication in the rhizosphere (Guttman et al., 2014) but still we do not have sufficient knowledge to comprehend the evolutionary origins and stability of the rhizosphere communication system. Comprehension of major beneficial plant–microbe interactions such as arbuscular mycorrhizas and the plant growth promoting rhizobacteria (PGPR)–legume symbiosis have been changed over the past years. The PGPR–legume root symbiosis and arbuscular mycorrhizal (AM) symbioses are established by exchanging a number of signals as there is mutual identification of diffusible signal molecules generated from both plants and microbial partner. A common symbiotic pathway (CSP) is triggered by symbiotic signals produced by rhizospheric bacteria or fungi which are in form of lipo-chitooligosaccharides (LCOs). These LCOs are perceived via lysine-motif (LysM) receptors found on the plasma membrane of plant cell and activate the CSP which regulate the interactions between plant and rhizospheric microorganisms. LysM receptor families are found in both legume and non-legume plants and receive signals from both rhizobia (Nod factor signals) and AM fungi (Myc-LCO signals). A model of CSP triggered in plants has been described in **Figure 2** together with all the proteins and receptor molecules involved in signaling. Furthermore, in this review it has been tried to understand the signaling pathway among AM fungi and roots of their host plants, where organic food is exchanged for nutrients from soil. This symbiotic relationship is among the most prevalent and anticipated to have evolved roughly 450 Mya (Field et al., 2015). There are several evidences obtained that signaling pathways between AM fungi and roots of their plant hosts are so thriving that the components of this pathway have been recruited by plants to evolution of other symbiosis such as rhizobial N_2_-fixation (Geurts et al., 2012). Plants and microorganism use a signaling system to transmit information about their internal situation and their readiness for immigration or colonization, but how can these reach the desired recipients, and not others (Oldroyd, 2013). Theoretically, specific signaling is required at two levels a broader screening to identify or stimulating the mutualists and a finer screen, to distinguish high and low-quality strains within a mutualist microorganism (Werner and Kiers, 2015). Strigolactones are acting as a major plant signaling molecule in the symbiotic system of arbuscular mycorrhiza. Strigolactones are terpenoid lactones which are a byproduct of carotenoid metabolism (Bonfante and Genre, 2015). However, Strigolactones are plant hormones, which secondarily also act to attract AM fungi. Strigolactones act as a stimulus to initiate metabolic cycle of the AM fungus which promotes growth toward the roots (**Figure 3**; Gutjahr, 2014). The receptors for strigolactone in mycorrhizal fungi have not been yet discovered (Koltai, 2014) Different types of strigolactones have been emitted by different plants which vary from host to attract specific fungal species or strains (Conn et al., 2015). The germinating AM fungal spores were activated by strigolactones derived from a root which execute a series of signal molecules such as chitooligosaccharides and lipochitooligosaccharides. These signal molecules activate a set of reactions in the plant root system and consequently the cytosolic concentration of calcium boosts which further induces gene expression of activated AM fungi which directs to the creation of the pre-penetration apparatus. The reacting root will secrete cut-in monomers, signaling the fungi to form a hypopodium and initiate arbuscular growth (Padje et al., 2016). The PGPR is known to synthesize the phytohormones, auxins. Auxin production can occur via multiple pathways by both plants as well as PGPRs. There are certain papers available which report that indole-3-acetic acid (IAA) is a natural auxin acting as signaling molecules in microorganisms. IAA affects gene expression in some of microorganisms, thus IAA act as a reciprocal signaling molecule in microbe–plant interactions (Spaepen and Vanderleyden, 2011). The bacterial gene expression is regulated under the control of IAA has been first described for ipdC gene of *Azospirillum brasilense*. It has been reported that IAA act as an inhibitory signal molecule for viral gene expression by *Agrobacterium tumefaciens* a phytopathogen (Liu and Nester, 2006). Furthermore, auxin level in plant–PGPR interactions affects different levels of nodule formation in plants such as auxin transport inhibition by the flavonoids which act as indicators of specification of founder cell and auxins accumulations initiate the nodule formation and differentiation (Mathesius, 2008).

### *In silico* Methods in Understanding Interactions

**FIGURE 4:**
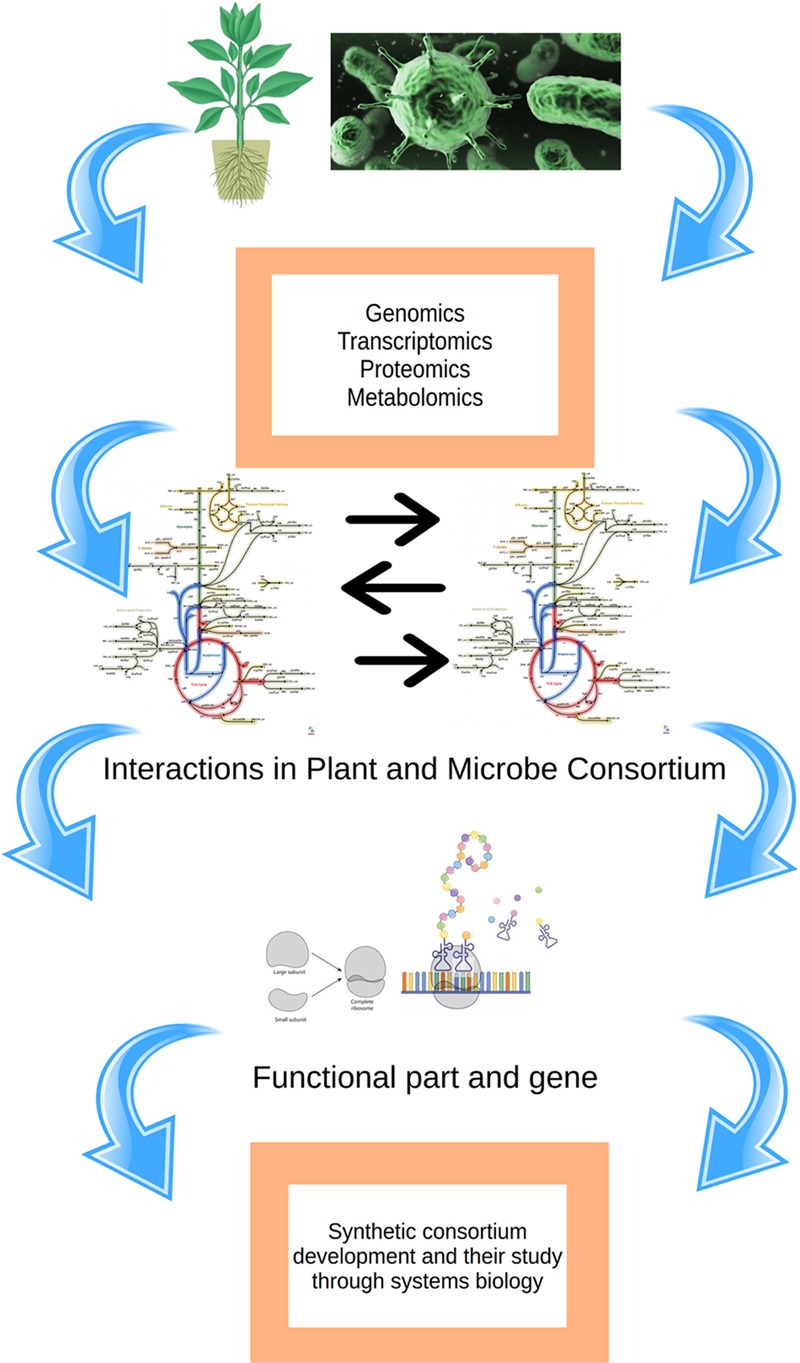
**An overview of the prerequisite for understanding plant–microbe interaction through systems biology**.

Systems biology is the study of genes, proteins and their interaction within a cell, tissue or whole organism. It also enables us to understand complex biological system and modeling it with the help of computational techniques. The interaction of host and pathogen in plants plays an important role in enhancing signaling cascade which brings change in the protein and eventually in the phenotypic expression. There are few notable studies on systems biology and molecular modeling tools to understand the microbial enzymes and similar proteins, but it lacks any further scope for studies of proteins involved in plant–microbe interaction (Singh and Shukla, 2011, 2015; Karthik and Shukla, 2012; Baweja et al., 2015, 2016; Singh et al., 2016). The study of *in silico* transcriptomes of both host and pathogen during the infection will contribute to the knowledge of changes occurring during the infection. There are different database which is dedicated to host–pathogen interaction. There is dynamic complexity in the plant–microbe interaction which occurs since edges represent processes in biological networks that may take time to occur and are dependent on the other factors in the network. Concentrations of metabolites in metabolic and signaling processes vary over time thus there could be several ways to model this time-dependent variation. Ordinary differential equations are employed for the analysis and calculation of biochemical process for metabolic kinetics studies. In such studies edges and node forms the complex, edges are associated with some value of parameters such as binding coefficients. Edges comprise of values representing a quantity or concentration. There are variations in the value of nodes over the time as the substrate is utilized or byproduct is formed. Flux is the rate at which material flows, flux is associated with the edges and carry a certain value. Understanding flux and managing it helps in the regulating the biological process dynamics. The study of the dynamic behavior of interaction is complex to analyze even studying a small, dynamic behavior requires certain parameters and information which requires multiple dimension overview. The networks and their dynamic characteristics may be significant and these processes should be confirmed with valid experimental models. Topologies related to metabolomics of cell are dynamic between the compartments and they change over the time. It is obvious to mention here that concentrations (or counts) of active proteins, crucial metabolites within the interacting cell are more inconsistent than the topology of the metabolic model. This indicates a clear overview about that existence and these factors define the network topology. Furthermore, the amount of each active element in such system has varied significantly so such attributes are accessed by metabolomics, transcriptomics, and proteomics and these can be taken as significant markers to explicit the host–microbial interactions. There are examples in which microbes dominated the over the molecular control of the host and resulted in exceptional results including production of “zombie ants” and mimicry of flowers by the fungi *Ophiocordyceps unilateralis* (Pontoppidan et al., 2009) and *Puccinia monoica* (Roy, 1994). Such examples exemplify the potential of microorganisms to control elegantly the physiological processes in host cells. In it quite important to mention here that such microbes have developed the capacity toward environment control and influence the surrounding factors. The systems biology approach helps to find out various ways toward the alteration of host plant cells. There are not many chances that all the symptoms that appear in the plant–microbe interaction come out as a disease, it is just the coincidental part that occurs. All pathogens are causing disease will not be the right thing to consider. The pathogens which attack the host first explore the most vulnerable element of the host network that could cause more disruption in the most economical way. By virtue of this, the host also develops its defense system and the pathogen attack may be detected only in those parts of the system which are structurally most responsive to these changes. Further, it is to mention here that host cell will be benefited because the reduction in the number of receptor and recognition proteins. Systems biology approach and mathematical modeling of the system could also lead us to develop novel strategies to control the disease. Apart from these, the metabolism of plant engineered in microbe will show the way to the production of different essential components which are commercially important such as fuel and pharmaceutical molecules. An overall depiction of the methods described above is given in **Figure 4**.

### Systems Biology Techniques for Deciphering Plant–Microbe Interaction

Metabolic engineering in microorganisms has been employed in different areas such as industrial microbiology, medical microbiology, and agricultural microbiology (Chotani et al., 2000; Nakamura and Whited, 2003). The targeted motive of metabolic engineering could be different, but the technology and platform remained unchanged. Recently, computational modeling emerged and changed the perspective to analyze metabolic engineering. Computational modeling anticipates the effect of genetic manipulations on metabolism, however, these methods need enzyme kinetic information that is still mostly unknown (Tepper and Shlomi, 2010). Constraint-based modeling (CBM), is an alternate which overcome these problems by examining the function of metabolic networks by relying on physical–chemical constraints (Price et al., 2003). There are certain genome-scale network models available for many microorganisms (Förster et al., 2003; Reed et al., 2003; Duarte et al., 2004). CBM has proved to be successful for large-scale microbial networks which involve metabolic engineering studies for different applications. A metabolic reconstruction is a well-structured description of the network topology that enables derivation of genome-scale models (GEMs) that are used to mimic different metabolic states of an organism (Satish Kumar et al., 2007; Thiele and Palsson, 2010; Esvelt and Wang, 2013). Such technology has gained popularity for systems biology studies as it enables the integration of omics and overall analysis to explore the interplay of metabolic networks (Saha et al., 2014). A few metabolic reconstructions have been developed for different plant species, including *Arabidopsis* (Poolman et al., 2009; de Oliveira Dal’Molin et al., 2010a), maize (de Oliveira Dal’Molin et al., 2010b; Saha et al., 2011), sugarcane, and sorghum (deOliveira Dal’Molin et al., 2010b). The effectors act outside the host cell and sometimes secrete small molecules that may affect the host and modifies its biochemistry, for example, coronatine. We understand systems biology perspectives can be well applied to study such effectors and their pathogenesis aspects. These studies are based on certain tools which help in analyzing large amount of genomic data, interactions, GEMs this is depicted in **Table 1**. OptKnock is a technique which searches for sets of gene knockouts that lead to the production of desired products (Burgard et al., 2003) and can be used for the same purpose which can resist the plant from harmful microbial compounds. On the other hand, OptStrain that not only allows gene knockouts, but also incorporate novel enzyme-coding genes from different species to a given microbial genome (Pharkya et al., 2004). More recently, OptReg was developed, searching for manipulations in the form of up- and down-regulation of metabolic enzymes in addition to gene knockouts to meet desired metabolite production (Pharkya and Maranas, 2006).

**Table 1:** Applications of tools related to systems biology.

Name	Description	Operating system	License
BioTapestry	Interactive tool for building, visualizing, and simulating genetic regulatory networks	Multiplatform (Java-based)	LGPL
Cytoscape	Data integration, network visualization, and analysis	Multiplatform (Java-based)	LGPL
GenMAPP	Visualize and analyze genomic data in the context of pathways	Windows	Apache License
MEGA	Free, online, open-source, phylogenetic analysis, drawing dendrograms, etc.	Windows/DOS-Win/Mac/Linux	Shareware
PathVisio	Tool for displaying and editing biological pathways	Multiplatform (Java-based)	Apache License
InCroMAP	Tool for the integration of omics data and joint visualization of experimental data in pathways	Multiplatform (Java-based)	LGPL
Pathview	Pathway-based data integration and visualization, easy to use and integrate into pathway analysis	Multiplatform (R/Bioconductor)	GPL
Cell Designer	Structured diagram editor for gene-regulatory networks	Windows/Linux	The Systems Biology Institute, Tokyo, Japan (SBI, Japan)
Complex Pathway Stimulator (COPASI)	Simulation and analysis of biochemical networks	Windows/Linux	The Perl foundation
SBML toolbox	Analysis of SBML models in MATLAB	Windows/Linux	California Institute of Technology, Pasadena, CA, USA; EMBL European Bioinformatics Institute (EMBL-EBI), Hinxton, UK

### Gene Editing: An Approach to Develop Customized Functions

The recombinant DNA technology has revolutionized the study of the genome to a next level to provide the opportunity for its application in various fields like agriculture, industries, etc. The techniques like gene editing are proving as potential techniques in improvement of crop characters such as enhancing yield, providing resistance from biotic and abiotic stress. This has been possible because of major gene editing tools like zinc finger nucleases (ZFNs), transcription activator-like effector nucleases (TALEN), and clustered regularly interspaced short palindromic repeats (CRISPR-Cas) that introduce double strand break (DSB) in the target gene, which are repaired by the error-prone non-homologous end joining (NHEJ) pathway or homology-directed repair (HDR; Symington and Gautier, 2011).

ZFNs are artificial restriction enzymes that edit or cleave the specific target DNA by using zinc finger DNA-binding domain. The recognizing sequences *viz*. zinc finger domains can be artificially engineered to target specific sequences in the host. It consists of two DNA binding domains, the domain one is comprised of eukaryotic transcription factors and contain a zinc finger. The second domain includes the catalytic component, the nuclease *Fok*I restriction enzyme that catalyzes the specific DNA sequences. ZFNs have successfully performed well in defining the functions of various genes from diverse organism, including proven highly valuable in defining the roles of numerous genes in cells from a variety of organisms, including fruit flies, humans, mice, and higher plants (Gaj et al., 2013). However, there are certain drawbacks of ZHN technology like difficulties in design, construction, cost, and uncertain success rates.

TALEN are restriction enzymes that cleave target DNA by utilizing TAL effector DNA binding domains. The specific targeting is aided by simple “code” that matches with the di-amino acid sequence (repeat-variable di-residue) in ∼33–35 amino acid conserved target sequence. The progress in gene editing tools and development of various methods for easy synthesis and assembly of TALENs, allows the efficient editing at multiple sites. There have been various examples of the success of TALENs like knockout of the CCR5 gene for HIV resistance in human cells (Mussolino et al., 2011); destruction of the bacterial blight disease susceptibility gene in rice (Li et al., 2012); disruption of the LDL receptor in swine (Carlson et al., 2012); replacement of a tyrosine hydroxylase gene via TALEN-enhanced homologous recombination in zebrafish (Xiao et al., 2013; Zu et al., 2013).

### CRISPR-Cas in Understanding Interactions

Gene editing has been highly appreciated for their ability to change the desired DNA fragment using engineered nucleases often called as molecular scissors. Since it edits the product according to fitment of the process it has various applications in a diversity of areas. The CRISPR-Cas system has been evidenced as most efficient, easy and simple (Kanchiswamy et al., 2016). CRISPR-Cas system, also known as third-generation programmable nuclease has a major role in crop protection. There are approximately 11 CRISPR-Cas systems have been reported. They can be distinguished into three types (Types I–III) which are further divided into 11 subtypes (Ma and Liu, 2016). Each type has its own specific Cas protein component which is named according to model organism.

**FIGURE 5:**
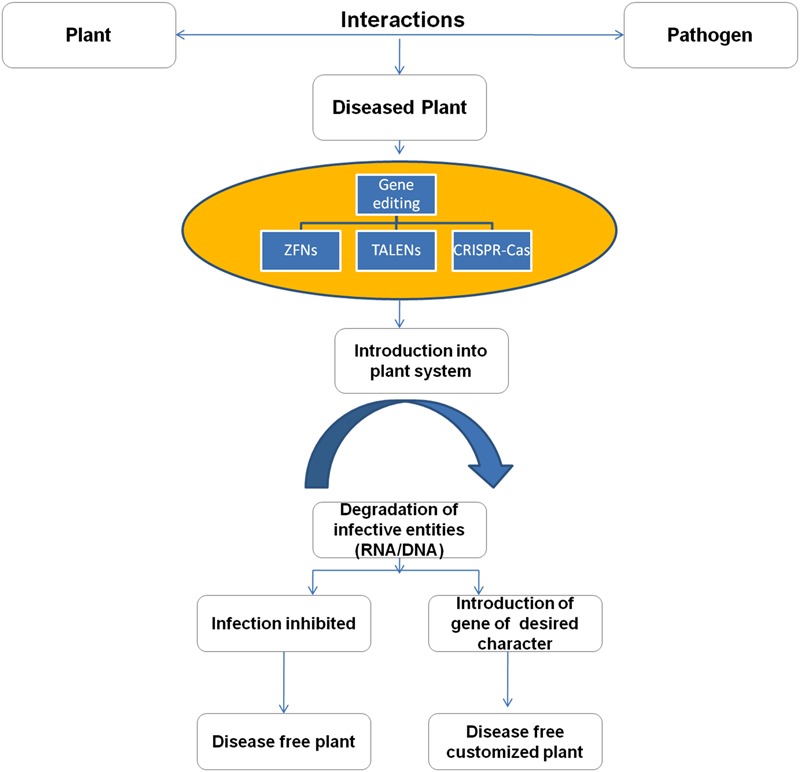
**Strategy of developing disease free plants using gene editing tools**.

Cas9 is a DNA endonuclease guided by RNA to target foreign DNA for inhibition (**Figure 5**) The guide RNAs (gRNAs) are derived from CRISPRs. CRISPRs consists of tandem arrays of a 30–40 bp short, direct repeat sequence which are separated by spacer sequences that matches the foreign sequence. Further transcription and processing of CRISPR produces mature CRISPR (cr)RNAs, the sequence flanked by signature CRISPR repeat tag at 5′ and 3′ end. The CRISPR (cr)RNAs form complex with Cas proteins to form a ribonucleoprotein (crRNP) that introduce cleavage in the DNA/RNA of the invader (Hale et al., 2012). One of the remarkable features of CRISPR is the specificity, that is aided by gRNA, that allows specific binding to target DNA and beauty of the system lies in the customized engineering of the gRNA. The specificity was enhanced by using double nickase and Cas9-nuclease fusion systems. Double nickase system allows binding of two gRNAs, both upstream as well as downstream preventing off target editing. This was further improved by using inactivated Cas9, i.e., without nuclease activity, fused with restriction enzymes. The nuclease activity of restriction enzyme only gets activated when both are in close proximity (Guilinger et al., 2014). The gene of interest can be inserted or deleted from the system with the help of CRISPR/Cas9 by introducing DSBs into a target site (Vanamee et al., 2001; Auer et al., 2014). Suitable expression construct is required for successful accomplishment of CRISPR-Cas sgRNA sequence(s), the codon-optimized variant of Cas9, strong promoters suitable to derive transcription of sgRNA and Cas9 (Raitskin and Patron, 2016). The importance of all these parameters was elucidated in a review by Schaeffer and Nakata (2015). With progress in computational techniques various computational tools like E-CRISP, CRISPR design tool, and CHOPCHOP have been developed that allow to identify the probable sequence of cleavage using input target sequences. Therefore, it helps to design gRNA (Hsu et al., 2013; Heigwer et al., 2014; Montague et al., 2014).

Once the target site is recognized by the gRNA, the nuclease Cas9 with the aid of its two domains RucV and HNH breaks the strand and generate blunt end DSB. Such DSB can be repaired by NHEJ that introduce mutation at the targeted site or by HDR, that may knock-in or replace the desired gene fragment at the target site using template DNA. There are various examples of gene editing utilized by different microbes (**Table 2**). Additionally, multiple editing in the same cell is possible using multiple gRNA that show various applications, like mutation in genes which are functionally related to control complex traits (Ma et al., 2015; Xie et al., 2015). In a study, expression of Cas9 and sgRNA genes in *Arabidopsis* and tobacco, caused a targeted cleavage of a non-functional GFP gene. Further mutation by NHEJ DNA repair led to the production of a strong green fluorescence in transforming leaf cells (Jiang et al., 2013, 2014).

**Table 2:** Genome editing in different plant species by the CRISPR/Cas technology.

Species	Transient/transgenic	Editing type	Delivery method	Off-target	Reference
*Arabidopsis thaliana*	Transient	NHEJ, HDR	Protoplast transfection	Not detected	Li et al., 2013
Lettuce (*Lactuca sativa*)	Transgenic	NHEJ	Protoplast transfection	Not detected	Woo et al., 2015
Barley (*Hordeum vulgare*)	Transgenic	NHEJ	*Agrobacterium*-mediated	Detected	Lawrenson et al., 2015
*Nicotiana attenuate*	Transgenic	NHEJ	Protoplast transfection	NA	
*Arabidopsis thaliana*	Transient	NHEJ	*Agrobacterium*-mediated	NA	Jiang et al., 2013
*Medicago truncatula*	Transgenic	NHEJ	*Agrobacterium*-mediated	NA	Michno et al., 2015

To enhance the expression of Cas9 in plants, codon optimization is often used strategically (Fauser et al., 2014). For the expression of Cas9, constitutive promoters of ubiquitin genes of rice, *Arabidopsis*, and maize can attain the desired requirement of gene editing in monocot and dicot plants.

### Plant–Virus Interactions and Desired Trait Improvement

Earlier, the studies on trait improvement were based on plant breeding, somatic hybridization, and random mutagenesis, the process was tedious and time consuming. The trend of plant breeding was replaced by efficient and simple tools, i.e., CRISPR-Cas to introduce specific traits into the population. The effort was done to enhance the sensitivity toward the herbicide. The three oligonucleotides were targeted by CRISPR-Cas via *A. tumefaciens*. The transformation was done using single gRNA in a binary vector and successfully mutants were found to be sensitive to bentazon herbicide. A genome modification study was done for the first time in the maize utilizing TALENs and CRISPR-Cas and concluded that both the systems efficiently can be used for genome modification in maize (Liang et al., 2014). Similar studies were done in tobacco and it also suggested that CRISPR-Cas is an efficient genome modification tool (Gao et al., 2015). The studies were done to enhance the gene targeting and it was observed that virus mediated transformation showed a higher frequency than the traditional *A. tumefaciens* T-DNA (Xu et al., 2014). Baltes et al. (2014) reported such finding in *Nicotiana tabacum* by using Gemini virus replicons to enhance the gene targeting and also revealed the DNA sequence editing using Gemini virus replicons. There have been a number of strategies for multiple gene targeting using multiple gRNA in a single plasmid vector described by Raitskin and Patron (2016). The Cas9 are now recently used to control the pests. In a study, the Cas9 was used to control the population of *Drosophila melanogaster*. Engineered endonuclease-based drive systems have been used to drive mutations into populations of pest species leading directly or indirectly to reduce population sizes (Reid and O’Brochta, 2016).

In near future, it is expected that CRISPR-Cas will prove as a remarkable tool to engineer plants to eradicate problems associated with crops like low yields, nutritional content, and resistance from biotic and abiotic factors. The technique can also be utilized to prevent the plant diseases by inhibiting the virus interaction with the plant system (**Figure 5**). The bacterial CRISPR-Cas could be used to inhibit the viral genetic material with the action of Cas9 as a nuclease thereby curtailing the establishment of viral infection in the plant (Ali et al., 2015; Baltes et al., 2015; Chaparro-Garcia et al., 2015; Ji et al., 2015). There are various examples where CRISPR-Cas system has proved to be successful in improving plant traits. In a rice plant, genetic modification was done in large chromosomal segments of sugar eﬄux transporter genes that resulted in 87–100% editing in T0 transgenic plants (Zhou et al., 2014). The gene function was first time revealed in the citrus fruit with the aid of CRISPR-Cas (Jia and Wang, 2014). CRISPR/Cas9 technology is most useful in woody plants that have long reproductive cycles, as they have the ability to acquire mutants in T0 generation (Fan et al., 2015; Tsai and Xue, 2015). Indeed, such results of gene editing empower the idea of the customized editing and desired expression in all living systems.

Certainly, successful development of the Cas9/sgRNA system for targeted gene modification and genome editing holds promise for boosting fundamental knowledge of plant biology as well as for designing crop plants with potential new agronomic, nutritional, and novel traits for the benefit of farmers and consumers.

## Conclusion

Microbes play a fundamental role in diverse ecosystems through microbial interactions with other biotic and abiotic components of the ecosystem. Plant–microbe interactions play an important role in plant health and ecological sustainability. So, comprehension of these interactions is very crucial to improve plant health and ecological sustainability. Recently, microbial interaction prediction using computational biology has become an extensively used approach to inspect the plant–microbial interactions. In this review, different computational methods developed by the computational data has been summarized to understand plant–microbe interactions. Several systems biology tools such as FBA (flux balance analysis), CBM, and OptKnock has been described to understand the metabolic pathways involved in plant–microbe interactions. Furthermore, gene editing tools such as TALENs and CRISPER-Cas have been described to control the pathogen interactions with plants to obtain customized plants. A snapshot of gene editing tools has been described to obtain disease free customized plants. There should be a better understanding of signaling pathways and metabolic networks to have an understanding of plant–microbial interactions. A combinatorial approach of computational biology and genomic tools has proven supportive to understand the communication pathway and metabolic pathway and provides an alternative to regulate these pathways to get a beneficial effect on plants with ecological sustainability.

## Author Contributions

All authors listed, have made substantial, direct and intellectual contribution to the work, and approved it for publication.

## Conflict of Interest Statement

The authors declare that the research was conducted in the absence of any commercial or financial relationships that could be construed as a potential conflict of interest.

## References

[B1] Ali Z. Abulfaraj A. Idris A. Ali S. Tashkandi M. Mahfouz M. M. (2015). CRISPR/Cas9-mediated viral interference in plants. *Genome Biol.* 16:238 10.1186/s13059-015-0799-6 PMC4641396 26556628

[B2] Andreo-Jimenez B. Ruyter-Spira C. Bouwmeester H. Lopez-Raez J. (2015). Ecological relevance of strigolactones in nutrient uptake and other abiotic stresses and in plant-microbe interactions below-ground. *Plant Soil* 394 1–19. 10.1007/s11104-015-2544-z

[B3] Auer T. O. Duroure K. Cian A. D. Concordet J. P. Bene F. D. (2014). Highly efficient CRISPR/Cas9- mediated knock-in in zebrafish by homology-independent DNA repair. *Genome Res.* 24 142–153. 10.1101/gr.161638.113 24179142 PMC3875856

[B4] Bakker P. A. H. M. Berendsen R. L. Doornbos R. F. Wintermans P. C. A. Pieterse C. M. J. (2013). The rhizosphere revisited: root microbiomics. *Front. Plant Sci.* 4:165 10.3389/fpls.2013.00165 PMC3667247 23755059

[B5] Baltes N. J. Hummel A. W. Konecna E. Cegan R. Bruns A. N. Bisaro D. M. (2015). Conferring resistance to geminiviruses with the CRISPR-Cas prokaryotic immune system. *Nat. Plants* 1:15145 10.1038/nplants.2015.145 PMC8612103 34824864

[B6] Baltes N. J. Javier G. H. Tomas C. Paul A. A. Daniel V. F. (2014). DNA replicons for plant genome engineering. *Plant Cell* 26 151–163. 10.1105/tpc.113.119792 24443519 PMC3963565

[B7] Bardgett R. D. van der Putten W. H. (2014). Belowground biodiversity and ecosystem functioning. *Nature* 515 505–511. 10.1038/nature13855 25428498

[B8] Bauer W. D. Mathesius U. (2004). Plant responses to bacterial quorum sensing signals. *Curr. Opin. Plant Biol.* 7 429–433. 10.1016/j.pbi.2004.05.008 15231266

[B9] Baweja M. Nain L. Kawarabayasi Y. Shukla P. (2016). Current Technological Improvements in Enzymes towards their biotechnological applications. *Front. Microbiol.* 7:965 10.3389/fmicb.2016.00965 PMC4909775 27379087

[B10] Baweja M. Singh P. K. Shukla P. (2015). “Enzyme technology, functional proteomics and systems biology towards unraveling molecular basis for functionality and interactions in biotechnological processes,” in *Frontier Discoveries and Innovations in Interdisciplinary Microbiology* ed. Shukla P. (Heidelberg: Springer-Verlag) 207–212.

[B11] Bonfante P. Genre A. (2015). Arbuscular mycorrhizal dialogues: do you speak ‘plantish’ or ‘fungish’? *Trends Plant Sci.* 20 150–154. 10.1016/j.tplants.2014.12.002 25583176

[B12] Buffie C. G. Bucci V. Stein R. R. McKenney P. T. Ling L. Gobourne A. (2014). Precision microbiome reconstitution restores bile acid mediated resistance to *Clostridium difficile*. *Nature* 517 205–208. 10.1038/nature13828 25337874 PMC4354891

[B13] Burgard A. P. Pharkya P. Maranas C. D. (2003). Optknock: a bilevel programming framework for identifying gene knockout strategies for microbial strain optimization. *Biotechnol. Bioeng.* 84 647–657. 10.1002/bit.10803 14595777

[B14] Campbell N. (1995). *Prokaryotes and the Origins of Metabolic Diversity* 5th Edn eds Brady E. B. (Reedwood City, CA: The Benjamin/Cummings Publishing Company) 502–519.

[B15] Carlson D. F. Tan W. Lillico S. G. Stverakova D. Proudfoot C. Christian M. (2012). Efficient TALEN-mediated gene knockout in livestock. *Proc. Natl. Acad. Sci. U.S.A.* 43 17382–17387. 10.1073/pnas.1211446109 23027955 PMC3491456

[B16] Castro-Sowinski S. Herschkovitz Y. Okon Y. Jurkevitch E. (2007). Effects of inoculation with plant growth-promoting rhizobacteria on resident rhizosphere microorganisms. *FEMS Microbiol. Lett.* 276 1–11. 10.1111/j.1574-6968.2007.00878.x 17711454

[B17] Chaparro-Garcia A. Kamoun S. Nekrasov V. (2015). Boosting plant immunity with CRISPR/Cas. *Genome Biol.* 16:254 10.1186/s13059-015-0829-4 PMC4653885 26585913

[B18] Chotani G. Dodge T. Hsu A. Kumar M. LaDuca R. Trimbur D. (2000). The commercial production of chemicals using pathway engineering. *Biochim. Biophys. Acta* 1543 434–455. 10.1016/S0167-4838(00)00234-X 11150618

[B19] Conn C. E. Bythell-Douglas R. Neumann D. Yoshida S. Whittington B. Westwood J. H. (2015). Convergent evolution of strigolactone perception enabled host detection in parasitic plants. *Science* 349 540–543. 10.1126/science.aab1140 26228149

[B20] Cornforth D. M. Popat R. McNally L. Gurney J. Scott-Phillips T. C. Ivens A. (2014). Combinatorial quorum sensing allows bacteria to resolve their social and physical environment. *Proc. Natl. Acad. Sci. U.S.A.* 111 4280–4284. 10.1073/pnas.1319175111 24594597 PMC3964068

[B21] de Oliveira Dal’Molin C. G. Quek L. E. Palfreyman R. W. Brumbley S. M. Nielsen L. K. (2010a). AraGEM, a genome-scale reconstruction of the primary metabolic network in *Arabidopsis*. *Plant Physiol.* 152 579–589. 10.1104/pp.109.148817 20044452 PMC2815881

[B22] de Oliveira Dal’Molin C. G. Quek L. E. Palfreyman R. W. Brumbley S. M. Nielsen L. K. (2010b). C4GEM, a genome-scale metabolic model to studyC4 plant metabolism. *Plant Physiol.* 154 1871–1885. 10.1104/pp.110.166488 20974891 PMC2996019

[B23] Dix A. Vlaic S. Guthke R. Linde J. (2016). Use of systems biology to decipher host-pathogen interaction networks and predict biomarkers. *Clin. Microbiol. Infect.* 22 600–606. 10.1016/j.cmi.2016.04.014 27113568

[B24] Duarte N. C. Herrgård M. J. Palsson B. Ø. (2004). Reconstruction and validation of saccharomyces cerevisiae ind750, a fully compartmentalized genome-scale metabolic model. *Genome Res.* 14 1298–1309. 10.1101/gr.2250904 15197165 PMC442145

[B25] Esvelt K. M. Wang H. H. (2013). Genome-scale engineering for systems and synthetic biology. *Mol. Syst. Biol.* 9:641 10.1038/msb.2012.66 PMC3564264 23340847

[B26] Fan D. Liu T. Li C. Jiao B. Li S. Hou Y. (2015). Efficient CRISPR/Cas9-mediated targeted mutagenesis in Populus in the first generation. *Sci. Rep.* 5:12217 10.1038/srep12217 PMC4507398 26193631

[B27] Fauser F. Schiml S. Puchta H. (2014). Both CRISPR/Cas-based nucleases and nickases can be used efficiently for genome engineering in *Arabidopsis thaliana*. *Plant J.* 79 348–359. 10.1111/tpj.12554 24836556

[B28] Field K. J. Pressel S. Duckett J. G. Rimington W. R. Bidartondo M. I. (2015). Symbiotic options for the conquest of land. *Trends Ecol. Evol.* 30 477–486. 10.1016/j.tree.2015.05.007 26111583

[B29] Fillion M. (2008). Do transgenic plants affect rhizobacteria populations? *Microb. Biotechnol.* 1 463–475. 10.1111/j.1751-7915.2008.00047.x 21261867 PMC3815288

[B30] Förster J. Famili I. Fu P. Palsson B. Ø. Nielsen J. (2003). Genome-scale reconstruction of the saccharomyces cerevisiae metabolic network. *Genome Res.* 13 244–253. 10.1101/gr.234503 12566402 PMC420374

[B31] Freilich S. Zarecki R. Eilam O. Segal E. S. Henry C. S. Kupiec M. (2011). Competitive and cooperative metabolic interactions in bacterial communities. *Nat. Commun.* 2:589 10.1038/ncomms1597 22158444

[B32] Gaj T. Gersbach C. A. Barbas C. F. (2013). ZFN, TALEN, and CRISPR/Cas-based methods for genome engineering. *Trends Biotechnol.* 31 397–405. 10.1016/j.tibtech.2013.04.004 23664777 PMC3694601

[B33] Gao J. Wang G. Ma S. Xie X. Wu X. Zhang X. (2015). CRISPR/Cas9-mediated targeted mutagenesis in *Nicotiana tabacum*. *Plant Mol. Biol.* 87 99–110. 25344637 10.1007/s11103-014-0263-0

[B34] Gao Z. Zhuang J. Chen J. Liu X. Tang S. (2004). Population of endophytic bacteria in maize roots and its dynamic analysis. *Ying Yong Sheng Tai Xue Bao* 15 1344–1348. 15573985

[B35] Geurts R. Lillo A. Bisseling T. (2012). Exploiting an ancient signalling machinery to enjoy a nitrogen fixing symbiosis. *Curr. Opin. Plant Biol.* 15 438–443. 10.1016/j.pbi.2012.04.004 22633856

[B36] Gough C. Galera C. Vasse J. Webster G. Cocking E. C. Denarie J. (1997). Specific flavonoids promote intercellular root colonization of *Arabidopsis thaliana* by *Azorhizobium caulinodans* ORS571. *Mol. Plant Microbe Interact.* 10 560–570. 10.1094/MPMI.1997.10.5.560 9204562

[B37] Guilinger J. P. Thompson D. B. Liu D. R. (2014). Fusion of catalytically inactive Cas9 to FokI nuclease improves the specificity of genome modification. *Nat. Biotechnol.* 32 577–582. 10.1038/nbt.2909 24770324 PMC4263420

[B38] Gupta S. K. Shukla P. (2015a). Advanced technologies for improved expression of recombinant proteins in bacteria: perspectives and applications. *Crit. Rev. Biotechnol.* 18 1–10. 10.3109/07388551.2015.1084264 26384140

[B39] Gupta S. K. Shukla P. (2015b). Gene editing for cell engineering: trends and applications. *Crit. Rev. Biotechnol.* 10.1080/07388551.2016.1214557 [Epub ahead of print]. 27535623

[B40] Gupta S. K. Shukla P. (2016). Microbial platform technology for recombinant antibody fragment production. A review. *Crit. Rev. Microbiol.* 1–12. 10.3109/1040841X.2016.1150959 27387055

[B41] Gutjahr C. (2014). Phytohormone signaling in arbuscular mycorrhiza development. *Curr. Opin. Plant Biol.* 20 26–34. 10.1016/j.pbi.2014.04.003 24853646

[B42] Guttman D. S. McHardy A. C. Schulze-Lefert P. (2014). Microbial genome-enabled insights into plant-microorganism interactions. *Nat. Rev. Genet.* 15 797–813. 10.1038/nrg3748 25266034

[B43] Hale C. R. Majumdar S. Elmore J. Pfister N. Compton M. Olson S. (2012). Essential features and rational design of CRISPR RNAs that function with the Cas RAMP module complex to cleave RNAs. *Mol. Cell* 45 292–302. 10.1016/j.molcel.2011.10.023 22227116 PMC3278580

[B44] Harcombe W. (2010). Novel cooperation experimentally evolved between species. *Evolution* 64 2166–2172. 10.1111/j.1558-5646.2010.00959.x 20100214

[B45] Heigwer F. Kerr G. Boutros M. (2014). E-CRISP: fast CRISPR target site identification. *Nat. Methods* 11 122–123. 10.1038/nmeth.2812 24481216

[B46] Hsu P. D. Scott D. A. Weinstein J. A. Ran F. A. Konermann S. Agarwala V. (2013). DNA targeting specificity of RNA-guided Cas9 nucleases. *Nat. Biotechnol.* 31 827–832. 10.1038/nbt.2647 23873081 PMC3969858

[B47] Imam J. Alam S. Mandal N. P. Maiti D. Variar M. Shukla P. (2015a). Molecular Diversity and Mating Type distribution of the rice blast pathogen *Magnaporthe oryzae* in North-East and Eastern India. *Indian J. Microbiol.* 55 108–113. 10.1007/s12088-014-0504-6

[B48] Imam J. Alam S. Mandal N. P. Shukla P. Sharma T. R. Variar M. (2015b). Molecular identification and virulence analysis of AVR genes in rice blast pathogen, *Magnaporthe oryzae* from Eastern India. *Euphytica* 206 21–31. 10.1007/s10681-015-1465-5

[B49] Imam J. Alam S. Mandal N. P. Variar M. Shukla P. (2013a). Molecular screening for identification of blast resistance genes in North East and Eastern Indian rice germplasm (*Oryza sativa* L.) with PCR based makers. *Euphytica* 196 199–211. 10.1007/s10681-013-1024-x

[B50] Imam J. Alam S. Variar M. Shukla P. (2013b). Identification of rice blast resistance gene Pi9 from Indian rice land races with STS marker and its verification by virulence analysis. *Proc. Natl. Acad. Sci. U.S.A.* 83 499–504. 10.1007/s40011-013-0186-6

[B51] Imam J. Mahto D. Mandal N. P. Maiti D. Shukla P. Variar M. (2014). Molecular analysis of indian rice germplasm accessions with resistance to blast pathogen pages. *J. Crop Improv.* 28 729–739. 10.1080/15427528.2014.921261

[B52] Imam J. Variar M. Shukla P. (2013c). “Role of enzymes and proteins in plant-microbe interaction: a study of M. oryzae vs rice,” in *Advances in Enzyme Biotechnology* eds Shukla P. Pletschke B. I (Heidelberg: Springer-Verlag) 137–145.

[B53] James E. K. Olivares F. L. (1998). Infection and colonization of sugar cane and other graminaceous plants by endophytic diazotrophs. *Crit. Rev. Plant Sci.* 17 77–119. 10.1016/S0735-2689(98)00357-8

[B54] Ji X. Zhang H. Zhang Y. Wang Y. Gao C. (2015). Establishing a CRISPR-Cas-like immune system conferring DNA virus resistance in plants. *Nat. Plants* 1:15144 10.1038/nplants.2015.144 27251395

[B55] Jia H. Wang N. (2014). Targeted genome editing of sweet orange using Cas9/sgRNA. *PLoS ONE* 9:e93806 10.1371/journal.pone.0093806 PMC3977896 24710347

[B56] Jiang W. Brueggeman A. J. Horken K. M. Plucinak T. M. Weeks D. P. (2014). Successful transient expression of Cas9 and single guide RNA genes in *Chlamydomonas reinhardtii*. *Eukaryot. Cell* 13 1465–1469. 10.1128/EC.00213-14 25239977 PMC4248704

[B57] Jiang W. Zhou H. Bi H. Fromm M. Yang B. Weeks D. P. (2013). Demonstration of CRISPR/Cas9/sgRNA-mediated targeted gene modification in *Arabidopsis*, tobacco, sorghum and rice. *Nucleic Acids Res.* 41 e188 10.1093/nar/gkt780 PMC3814374 23999092

[B58] Kanchiswamy C. N. Maffei M. Malnoy M. Velasco R. Kim J. S. (2016). Fine-tuning next-generation genome editing tools. *Trends Biotechnol.* 34 562–574. 10.1016/j.tibtech.2016.03.007 27167723

[B59] Karthik M. V. K. Shukla P. (2012). *Computational Strategies Towards Improved Protein Function Prophecy of Xylanases From Themomyces lanuginosus.* New York, NY: Springer 10.1007/978-1-4614-4723-8

[B60] Kato S. Haruta S. Cui Z. J. Ishii M. Igarashi Y. (2005). Stable coexistence of five bacterial strains as a cellulose-degrading community. *Appl. Environ. Microbiol.* 71 7099–7106. 10.1128/AEM.71.11.7099-7106.2005 16269746 PMC1287685

[B61] Koltai H. (2014). Receptors, repressors, PINs: a playground for strigolactone signaling. *Trends Plant Sci.* 19 727–733. 10.1016/j.tplants.2014.06.008 25037847

[B62] Lawrenson T. Shorinola O. Stacey N. Li C. Ostergaard L. Patron N. (2015). Induction of targeted, heritable mutations in barley and *Brassica oleracea* using RNA-guided Cas9 nuclease. *Genome Biol.* 16:258 10.1186/s13059-015-0826-7 PMC4663725 26616834

[B63] Li J. F. Norville J. E. Aach J. McCormack M. Zhang D. Bush J. (2013). Multiplex and homologous recombinationmediated genome editing in *Arabidopsis* and *Nicotiana benthamiana* using guide RNA and Cas9. *Nat. Biotechnol.* 31 688–691. 10.1038/nbt.2654 23929339 PMC4078740

[B64] Li T. Liu B. Spalding M. H. Weeks D. P. Yang B. (2012). High-efficiency TALEN-based gene editing produces disease-resistant rice. *Nat. Biotechnol.* 30 390–392. 10.1038/nbt.2199 22565958

[B65] Liang Z. Zhang K. Chen K. Gao C. (2014). Targeted mutagenesis in *Zea mays* using TALENs and the CRISPR/Cas system. *J. Genet. Genomics* 41 63–68. 10.1016/j.jgg.2013.12.001 24576457

[B66] Lima-Mendez G. Faust K. Henry N. Decelle J. Colin S. Carcillo F. (2015). Determinants of community structure in the global plankton interactome. *Science* 348:1262073 10.1126/science.1262073 25999517

[B67] Liu P. Nester E. W. (2006). Indoleacetic acid, a product of transferred DNA, inhibits vir gene expression and growth of *Agrobacterium tumefaciens* C58. *Proc. Natl. Acad. Sci. U.S.A.* 103 4658–4662. 10.1073/pnas.0600366103 16537403 PMC1450227

[B68] Ma X. Liu Y. G. (2016). Crispr/cas9-based multiplex genome editing in monocot and dicot plants. *Curr. Protoc. Mol. Biol.* 1 115–131. 10.1002/cpmb.10 27366892

[B69] Ma X. Zhang Q. Zhu Q. Liu W. Chen Y. Qiu R. (2015). A robust CRISPR/Cas9 system for convenient, high-efficiency multiplex genome editing in monocot and dicot plants. *Mol. Plant* 8 1274–1284. 10.1016/j.molp.2015.04.007 25917172

[B70] Mathesius U. (2008). Auxin: at the root of nodule development? *Funct. Plant Biol.* 35 651–668. 10.1071/FP08177 32688821

[B71] Michno J. M. Wang X. Liu J. Curtin S. J. Kono T. J. Stupar R. M. (2015). CRISPR/Cas mutagenesis of soybean and *Medicago truncatula* using a new web-tool and a modified Cas9 enzyme. *GM Crops Food* 6 243–252. 10.1080/21645698.2015.1106063 26479970 PMC5033229

[B72] Miller J. B. Oldroyd G. D. (2012). “The role of diffusible signals in the establishment of rhizobial and mycorrhizal symbioses,” in *Signaling and Communication in Plant Symbiosis* Vol. 11 eds Perotto S. Baluška F. (Heidelberg: Springer) 1–30. 10.4161/psb.22894

[B73] Mokkonen M. Lindstedt C. (2015). The evolutionary ecology of deception. *Biol. Rev.* 10.1111/brv.12208 26118820

[B74] Montague T. G. Cruz J. M. Gagnon J. A. Church G. M. Valen E. (2014). CHOPCHOP: a CRISPR/Cas9 and TALEN web tool for genome editing. *Nucleic Acids Res.* 42 401–407. 10.1093/nar/gku410 PMC4086086 24861617

[B75] Mussolino C. Morbitzer R. Lütge F. Dannemann N. Lahaye T. Cathomen T. (2011). A novel TALE nuclease scaffold enables high genome editing activity in combination with low toxicity. *Nucleic Acids Res.* 21 9283–9293. 10.1093/nar/gkr597 21813459 PMC3241638

[B76] Nakamura C. E. Whited G. M. (2003). Metabolic engineering for the microbial production of 1,3-propanediol. *Curr. Opin. Biotechnol.* 14 454–459. 10.1016/j.copbio.2003.08.005 14580573

[B77] Oldroyd G. E. D. (2013). Speak, friend, and enter: signalling systems that promote beneficial symbiotic associations in plants. *Nat. Rev. Microbiol.* 11 252–263. 10.1038/nrmicro2990 23493145

[B78] Padje A. V. Whiteside M. D. Kiers E. T. (2016). Signals and cues in the evolution of plant–microbe communication. *Curr. Opin. Plant Biol.* 32 47–52. 10.1016/j.pbi.2016.06.006 27348594

[B79] Parks S. E. Cusano D. A. Stimpert A. K. Weinrich M. T. Friedlaender A. S. Wiley D. N. (2014). Evidence for acoustic communication among bottom foraging humpback whales. *Sci. Rep.* 4:7508 10.1038/srep07508 PMC4267198 25512188

[B80] Pharkya P. Burgard A. P. Maranas C. D. (2004). Optstrain: a computational framework for redesign of microbial production systems. *Genome Res.* 14 2367–2376. 10.1101/gr.2872004 15520298 PMC525696

[B81] Pharkya P. Maranas C. D. (2006). An optimization framework for identifying reaction activation/inhibition or elimination candidates for overproduction in microbial systems. *Metab. Eng.* 8 1–13. 10.1016/j.ymben.2005.08.003 16199194

[B82] Pontoppidan M. B. Himaman W. Hywel-Jones N. L. Boomsma J. J. Hughes D. P. (2009). Graveyards on the move: the spatio-temporal distribution of dead *Ophiocordyceps*-infected ants. *PLoS ONE* 4:4835 10.1371/journal.pone.0004835 PMC2652714 19279680

[B83] Poolman M. G. Miguet L. Sweetlove L. J. Fell D. A. (2009). A genome-scale metabolic model of *Arabidopsis* and some of its properties. *Plant Physiol.* 151 1570–1581. 10.1104/pp.109.141267 19755544 PMC2773075

[B84] Price N. D. Papin J. A. Schilling C. H. Palsson B. O. (2003). Genome-scale microbial in silico models: the constraints-based approach. *Trends Biotechnol.* 21 162–169. 10.1016/S0167-7799(03)00030-1 12679064

[B85] Pritchard L. Birch P. (2011). A systems biology perspective on plant-microbe interactions: biochemical and structural targets of pathogen effectors. *Plant Sci.* 180 584–603. 10.1016/j.plantsci.2010.12.008 21421407

[B86] Raitskin O. Patron N. J. (2016). Multi-gene engineering in plants with RNA-guided Cas9 nuclease. *Curr. Opin. Biotechnol.* 37 69–75. 10.1016/j.copbio.2015.11.008 26707469

[B87] Reed J. L. Vo T. D. Schilling C. H. Palsson B. O. (2003). An expanded genome-scale model of *Escherichia coli* K-12 (Ijr904 sm/Gpr). *Genome Biol.* 4:R54 10.1186/gb-2003-4-9-r54 PMC193654 12952533

[B88] Reid W. O’Brochta D. A. (2016). Applications of genome editing in insects. *Curr. Opin. Insect Sci.* 13 43–54. 10.1016/j.cois.2015.11.001 27436552

[B89] Roy B. A. (1994). The use and abuse of pollinators by fungi. *Trends Ecol. Evol.* 9 335–339. 10.1016/0169-5347(94)90154-6 21236877

[B90] Ryan R. P. Germaine K. Franks A. Ryan D. J. Dowling D. N. (2008). Bacterial endophytes: recent developments and applications. *FEMS Microbiol. Lett.* 278 1–9. 10.1111/j.1574-6968.2007.00918.x 18034833

[B91] Saha R. Chowdhury A. Maranas C. D. (2014). Recent advances in the Reconstruction of metabolic models and integration of omicsdata. *Curr. Opin. Biotechnol.* 29 39–45. 10.1016/j.copbio.2014.02.011 24632194

[B92] Saha R. Suthers P. F. Maranas C. D. (2011). *Zea mays* iRS1563: a comprehensive genome-scale metabolic reconstruction of maize metabolism. *PLoS ONE* 6:e21784 10.1371/journal.pone.0021784 PMC3131064 21755001

[B93] Satish Kumar V. Dasika M. S. Maranas C. D. (2007). Optimization based automated curation of metabolic reconstructions. *BMC Bioinformatics* 8:212 10.1186/1471-2105-8-212 PMC1933441 17584497

[B94] Schaeffer S. M. Nakata P. A. (2015). CRISPR/Cas9-mediated genome editing and gene replacement in plants: transitioning from lab to field. *Plant Sci.* 240 130–142. 10.1016/j.plantsci.2015.09.011 26475194

[B95] Seghers D. Wittebolle L. Top E. M. Verstraete W. Siciliano S. D. (2004). Impact of agricultural practices on the *Zea mays* L. endophytic community. *Appl. Environ. Microbiol.* 70 1475–1482. 10.1128/AEM.70.3.1475-1482.2004 15006768 PMC368402

[B96] Singh B. K. Millard P. Whiteley A. S. Murrell J. C. (2004). Unravelling rhizosphere-microbial interactions: opportunities and limitations. *Trends Microbiol.* 12 386–393. 10.1016/j.tim.2004.06.008 15276615

[B97] Singh P. K. Joseph J. Goyal S. Grover A. Shukla P. (2016). Functional analysis of the binding model of microbial inulinases using docking and molecular dynamics simulation. *J. Mol. Model.* 22 1–7. 10.1007/s00894-016-2935-y 26956120

[B98] Singh P. K. Shukla P. (2011). Molecular modeling and docking of microbial inulinases towards perceptive enzyme-substrate interactions. *Indian J. Microbiol.* 52 373–380. 10.1007/s12088-012-0248-0 23997327 PMC3460115

[B99] Singh P. K. Shukla P. (2015). Systems biology as an approach for deciphering microbial interactions. *Brief. Funct. Genomics* 14 166–168. 10.1093/bfgp/elu023 24994863

[B100] Sørensen J. Sessitsch A. (2007). “Plant-associated bacteria – lifestyle and molecular interactions,” in *Modern Soil Microbiology* 2nd Edn eds Elsas J. D. V. Jansson J. K. Trevors J. T. (Boca Raton, FL: CRC Press) 211–236.

[B101] Spaepen S. Vanderleyden J. (2011). Auxin and plant-microbe interactions. *Cold Spring Harb. Perspect. Biol.* 3:a001438 10.1101/cshperspect.a001438 PMC3062209 21084388

[B102] Symington L. S. Gautier J. (2011). Double-strand break end resection and repair pathway choice. *Annu. Rev. Genet.* 45 247–271. 10.1146/annurev-genet-110410-132435 21910633

[B103] Tepper N. Shlomi T. (2010). Predicting metabolic engineering knockout strategies for chemical production: accounting for competing pathways. *Bioinformatics* 26 536–543. 10.1093/bioinformatics/btp704 20031969

[B104] Thiele I. Palsson B. Ø. (2010). A protocol for generating a high quality genome-scale metabolic reconstruction. *Nat. Protoc.* 5 93–121. 10.1038/nprot.2009.203 20057383 PMC3125167

[B105] Tsai C. J. Xue L. J. (2015). CRISPRing into the woods. *GM Crops Food* 6 206–215. 10.1080/21645698.2015.1091553 26357840 PMC5033219

[B106] Vanamee E. S. Santagata S. Aggarwal A. K. (2001). FokI requires two specific DNA sites for cleavage. *J. Mol. Biol.* 309 69–78. 10.1006/jmbi.2001.4635 11491302

[B107] Werner G. D. A. Kiers E. T. (2015). Partner selection in the mycorrhizal mutualism. *New Phytol.* 205 1437–1442. 10.1111/nph.13113 25421912

[B108] West S. A. Fisher R. M. Gardner A. Kiers E. T. (2015). Major evolutionary transitions in individuality. *Proc. Natl. Acad. Sci. U.S.A.* 112 10112–10119. 10.1073/pnas.1421402112 25964342 PMC4547252

[B109] Woo J. W. Kim J. Kwon S. I. Corvalan C. Cho S. W. Kim H. (2015). DNA-free genome editing in plants with preassembled CRISPR-Cas9 ribonucleoproteins. *Nat. Biotechnol.* 33 1162–1164. 10.1038/nbt.3389 26479191

[B110] Xiao A. Wang Z. Hu Y. Wu Y. Luo Z. Yang Z. (2013). Chromosomal deletions and inversions mediated by TALENs and CRISPR/Cas in zebrafish. *Nucleic Acids Res.* 41 e141 10.1093/nar/gkt464 PMC3737551 23748566

[B111] Xie K. Minkenberg B. Yang Y. (2015). Boosting CRISPR/Cas9 multiplex editing capability with the endogenous tRNA-processing system. *Proc. Natl. Acad. Sci. U.S.A.* 112 3570–3575. 10.1073/pnas.1420294112 25733849 PMC4371917

[B112] Xu P. Bhan N. Koffas M. A. (2013). Engineering plant metabolism into microbes: from systems biology to synthetic biology. *Curr. Opin. Biotechnol.* 24 291–299. 10.1016/j.copbio.2012.08.010 22985679

[B113] Xu R. Li H. Qin R. Wang L. Li L. Wei P. (2014). Gene targeting using the *Agrobacterium tumefaciens*-mediated CRISPR-Cas system in rice. *Rice (N. Y.)* 7:5 10.1186/s12284-014-0005-6 PMC4052633 24920971

[B114] Zeidan A. A. Rådström P. van Niel E. W. J. (2010). Stable coexistence of two *Caldicellulosiruptor* species in a de novo constructed hydrogen-producing co-culture. *Microb. Cell Fact.* 9:102 10.1186/1475-2859-9-102 PMC3022713 21192828

[B115] Zhou H. Liu B. Weeks D. P. Spalding M. H. Yang B. (2014). Large chromosomal deletions and heritable small genetic changes induced by CRISPR/Cas9 in rice. *Nucleic Acids Res.* 42 10903–10914. 10.1093/nar/gku806 25200087 PMC4176183

[B116] Zu Y. Tong X. Wang Z. Liu D. Pan R. Li Z. (2013). TALEN-mediated precise genome modification by homologous recombination in zebrafish. *Nat. Methods* 4 329–331. 10.1038/nmeth.2374 23435258

